# Next generation community assessment of biomedical entity recognition web servers: metrics, performance, interoperability aspects of BeCalm

**DOI:** 10.1186/s13321-019-0363-6

**Published:** 2019-06-24

**Authors:** Martin Pérez-Pérez, Gael Pérez-Rodríguez, Aitor Blanco-Míguez, Florentino Fdez-Riverola, Alfonso Valencia, Martin Krallinger, Anália Lourenço

**Affiliations:** 1 Department of Computer Science, ESEI, University of Vigo, Campus As Lagoas, 32004 Ourense, Spain; 2 The Biomedical Research Centre (CINBIO), Campus Universitario Lagoas-Marcosende, 36310 Vigo, Spain; 3 SING Research Group, Galicia Sur Health Research Institute (ISS Galicia Sur), SERGAS-UVIGO, Vigo, Spain; 4 Department of Microbiology and Biochemistry of Dairy Products, Instituto de Productos Lácteos de Asturias (IPLA), Consejo Superior de Investigaciones Científicas (CSIC), Paseo Río Linares S/N 33300, Villaviciosa, Asturias Spain; 5 Life Science Department, Barcelona Supercomputing Centre (BSC-CNS), C/Jordi Girona 29-31, 08034 Barcelona, Spain; 6 Joint BSC-IRB-CRG Program in Computational Biology, Parc Científic de Barcelona, C/Baldiri Reixac 10, 08028 Barcelona, Spain; 7 Institució Catalana de Recerca i Estudis Avançats (ICREA), Passeig de Lluís Companys 23, 08010 Barcelona, Spain; 8 Spanish Bioinformatics Institute INB-ISCIII ES-ELIXIR, 28029 Madrid, Spain; 9 Biological Text Mining Unit, Structural Biology and Biocomputing Programme, Spanish National Cancer Research Centre, C/Melchor Fernández Almagro 3, 28029 Madrid, Spain; 10 Centre of Biological Engineering (CEB), University of Minho, Campus de Gualtar, 4710-057 Braga, Portugal

**Keywords:** Named entity recognition, Shared task, REST-API, TIPS, BeCalm metaserver, Patent mining, Annotation server, Continuous evaluation, BioCreative, Text mining

## Abstract

**Background:**

Shared tasks and community challenges represent key instruments to promote research, collaboration and determine the state of the art of biomedical and chemical text mining technologies. Traditionally, such tasks relied on the comparison of automatically generated results against a so-called Gold Standard dataset of manually labelled textual data, regardless of efficiency and robustness of the underlying implementations. Due to the rapid growth of unstructured data collections, including patent databases and particularly the scientific literature, there is a pressing need to generate, assess and expose robust big data text mining solutions to semantically enrich documents in real time. To address this pressing need, a novel track called “Technical interoperability and performance of annotation servers” was launched under the umbrella of the BioCreative text mining evaluation effort. The aim of this track was to enable the continuous assessment of technical aspects of text annotation web servers, specifically of online biomedical named entity recognition systems of interest for medicinal chemistry applications.

**Results:**

A total of 15 out of 26 registered teams successfully implemented online annotation servers. They returned predictions during a two-month period in predefined formats and were evaluated through the BeCalm evaluation platform, specifically developed for this track. The track encompassed three levels of evaluation, i.e. data format considerations, technical metrics and functional specifications. Participating annotation servers were implemented in seven different programming languages and covered 12 general entity types. The continuous evaluation of server responses accounted for testing periods of low activity and moderate to high activity, encompassing overall 4,092,502 requests from three different document provider settings. The median response time was below 3.74 s, with a median of 10 annotations/document. Most of the servers showed great reliability and stability, being able to process over 100,000 requests in a 5-day period.

**Conclusions:**

The presented track was a novel experimental task that systematically evaluated the technical performance aspects of online entity recognition systems. It raised the interest of a significant number of participants. Future editions of the competition will address the ability to process documents in bulk as well as to annotate full-text documents.

**Electronic supplementary material:**

The online version of this article (10.1186/s13321-019-0363-6) contains supplementary material, which is available to authorized users.

## Introduction

There is a pressing need to process systematically the rapidly growing amount of unstructured textual data, not only in the domain of chemistry or pharmacology but also by almost all areas of scientific knowledge [1]. In the case of medicinal chemistry and biomedicine, the literature and patent collections cover two of the most valuable sources of information. The use of text mining and natural language processing technologies are showing promising results to be able to unlock valuable information hidden in those natural language datasets. In order to promote the development of competitive language technology solutions, the two key instruments have been (1) the combination of Gold Standard datasets and (2) the shared tasks or community challenges. Gold Standard datasets or corpora are typically used to train, develop and evaluate (as a sort of ground of truth dataset) text-mining approaches, while shared tasks offer a competitive environment where different strategies or participating teams are evaluated through a common evaluation setting using the same metrics, datasets and annotation formats [2]. In this line, shared task settings were not only used to assess the quality of automatically generated results against human labels but were also explored to analyse issues related to the real-life practical usage of systems and their interactive insertion and adoption into data curation workflows [3]. However, the limited availability of large enough high-quality hand-crafted Gold Standard corpora is currently still one of the main bottlenecks for developing text mining components. To mitigate this issue, some recent attempts were made to explore alternative data annotation scenarios, such as collective tagging by humans through crowdsourcing, which nevertheless faces several issues like limited annotation quality when used for tasks that require deep domain expertise [4], or fusing automatically generated annotations returned by multiple systems into some sort of consensus or silver standard datasets, as was the case of the CALBC effort [5]. Beyond *quality* aspects, one of the main limitations of most shared tasks is the lack of direct access to the underlying participating systems or software. To address this situation, one potential benchmark setting is to require participating teams to submit or upload the used executable processing pipelines that generate automatic results [6]. This is known as *software submission*, as opposed to *run submission* and was used, for instance, in general, domain language technology shared tasks [7, 8].

Previous BioCreative competitions were also focused on run submissions, specifically community efforts have contributed to monitor and improve quality aspects of particular text mining components, such as named entity recognition tools for genes/proteins [9] or chemicals [10]. The detection of biomedical named entities is a basic building block required for more complex relation extraction tasks, and thus efforts have been made to build annotated resources for various entity types (i.e. used to generalize biomedical language concepts to higher level groups) to evaluate or train NER approaches [11]. The benefits in terms of quality when combining individual runs into some ensemble system, as well as the practical problems of accessibility derived from tracks organized through offline submissions settings, was already pointed out during early BioCreative shared tasks [12].

On the other hand, software submissions evaluation settings, although having clear benefits such as reproducibility or transparency, do also show considerable downsides under certain circumstances. For instance, in cases where the shared task requires the implementation of rather complex processing workflows and/or are data-heavy at the side of participating systems (i.e. require large gazetteers or language models), the use of software submissions might constitute a burden at the side of contributing teams as well as at the side of task organizers. Moreover, there are also legal issues that need to be taken into account, for instance, related to licensing and legal constraints due to code redistribution restrictions of a particular third party component or lexical resource. Finally, in case of commercial teams, distributing the actual software solution is often not an option and therefore hinders their participation and evaluation under such settings.

To address this scenario, web services represent a more decentralized technological strategy that constituting a solution that is, in principle, programming language and platform independent. Web services are particularly popular in bioinformatics and since life science databases due to their advantages in terms of reusability and they do not need installation, which makes them particularly attractive for less technically skilled users or users with a light computational infrastructure. The usage of web-services techniques to construct building interoperable text-mining workflows requires: (1) careful standardization of data exchange formats, (2) data type definitions and (3) naming convention specification. Exploratory efforts in this direction were carried out, including: (1) hackathons [13], (2) the establishment of projects to properly define ontologies for bioinformatics web service data types and methods together with the construction of centralized repositories for service discovery [14], (3) the BioC track at BioCreative V focused on data sharing and communication formats for interoperable text mining components and data annotation [15], and (4) the combination of individual services into a sort of a meta-service to empower comparison and ensemble services using the Unstructured Information Management Architecture (UIMA) under the U-Compare framework [16].

This increasing demand in being able to evaluate, compare, visualise and integrate multiple text mining systems in order to easily and effectively access to process natural language document collections was one of the main aims of the latest BioCreative initiatives. Thus, several tasks tried to promote submissions through the development of online text annotation servers (ASs) by participating teams [17–20]. In particular, the BioCreative Meta-Server was the first distributed prototype platform to request, retrieve, unify and visualise biomedical textual annotations [21], providing a unified interface to the input and output of the various protein–protein interaction extraction tools [22]. Despite the relevance of those previous efforts, several crucial aspects have not been sufficiently or only partially addressed, including: (1) continuous evaluation, (2) extraction of textual content from heterogeneous sources, (3) harmonisation of different biomedical text annotation types, as well as (4) visualisation and comparative assessment of automatic and manual annotations. These objectives motivated the proposal of a new experimental task for the BioCreative V.5 challenge, published in this special issue of the Journal of Cheminformatics, in addition to a more traditional NER evaluation track [23]. The BeCalm (Biomedical Annotation Metaserver)—Technical Interoperability and Performance of annotation Servers (TIPS) task was presented as a novel experiment focused on the technical aspects of making text-mining systems available and interoperable, as well as continuously evaluating the performance of participating ASs.

The present paper describes the motivation and general functioning of the TIPS task, as well as the support provided by the BeCalm metaserver infrastructure.

## Methods

This section presents the architectural design of the novel BeCalm metaserver and how this platform was utilised by the participants throughout the competition. Then, the TIPS task is presented along with its evaluation metrics and process.

Opposite to the previous prototype of the metaserver, BeCalm biomedical annotation metaserver supported the continuous evaluation of ASs performance as well as individual server monitoring by both the track organizers and the corresponding teams [24]. The ASs implemented a Representational State Transfer (REST) Application Programming Interface (API) that listens and responds to the requests made by the BeCalm metaserver, which acted as a central access point to those base services, delivering a harmonised interface to different biomedical NER algorithms. Therefore, the TIPS novel task was not restricted to a particular annotation type but attempted to expose both novels as well as existing systems harmoniously through robust and competitive web services, well-defined annotation formats and descriptive metadata types. Moreover, ASs could support any number of biomedical named entity types/classes as long as they held practical interest to biomedical applications (e.g. entity types as chemicals, genes or proteins). These ASs could be fully developed in-house or integrate/adapt third-party recognition software as building block components. Besides, participation was not restricted to specific methods, i.e. teams could participate through services relying on machine learning-based strategies, gazetteer/pattern look-up approaches, or both.

### The BeCalm metaserver platform

**Fig. 1:**
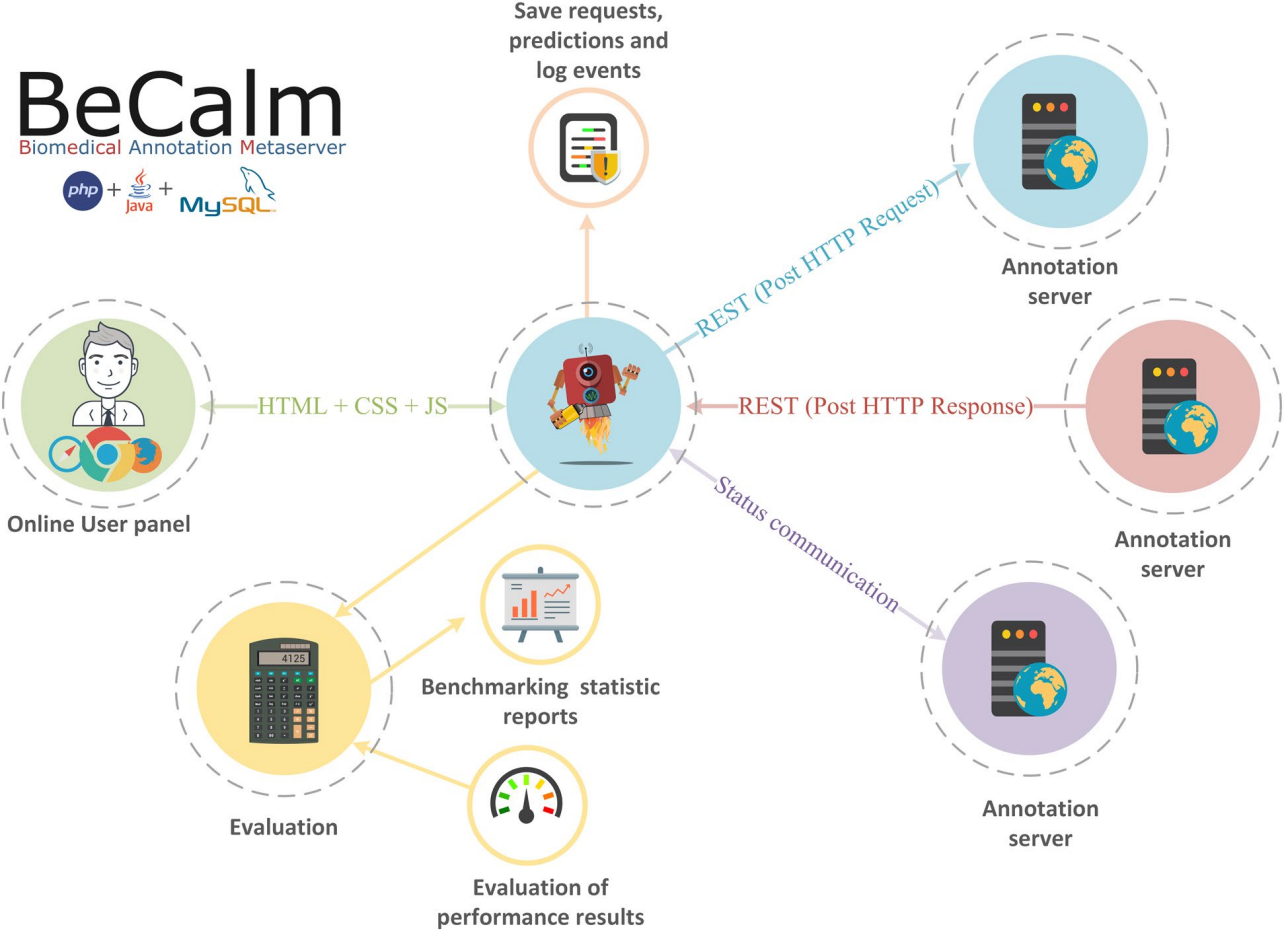
General overview figure to describe the BeCalm metaserver setting used for the TIPS track competition

The aspiration of the BeCalm biomedical annotation platform is to provide users with annotations of different kinds of biomedical and chemical texts gathered from different heterogeneous NER systems (Fig. 1). This novel platform was based on the design principles of simplicity, flexibility and expandability, offering a flexible API. To achieve this goal, we developed a platform consisting of a distributed system that requests and retrieves textual annotations from multiple online services, to further deliver the user different levels of customization to unify the data.

A few years ago, a first prototype of metaserver was developed [21]. This prototype was only focused on being a central point for obtaining biomedical annotations, while BeCalm is also able to objectively evaluate the capabilities of the online systems in terms of performance and stability. In this line, BeCalm implements and proposes several novel metrics and methodologies to evaluate the ASs. Furthermore, this perspective seeks to encourage that each developer may propose their biomedical entity types to cover an ever-increasing range of possibilities.

The BeCalm back-end was implemented using the open source CakePHP framework [25] and Java [26]. Whereas the BeCalm front-end was developed using mainstream Web user-system interaction technologies, such as HTML5 [27], CSS3 [28], Ajax and JQuery [29].

In order to robustly host the metaserver services, the in-house developed back-end is organised as a modular structure. This allows having two machine-independent services for managing the requests and responses. The first service is dedicated to the storage and evaluation of responses using a PHP REST API module [30]. The second service is a scheduler developed using Java and Hibernate ORM [31] and it is in charge of the creation and management of the annotation request process. Therefore, this scheduler is responsible for assembling and sending the batch processing requests to the different ASs at a certain daytime, supporting regular and irregular request time windows.

This second service sends annotation requests to all registered ASs and then the PHP REST API of the first service saves the result and the meta-information (i.e. response time, NER types returned or the number of predictions) of those ASs who return predictions (considering various biomedical annotation types).

**Fig. 2:**
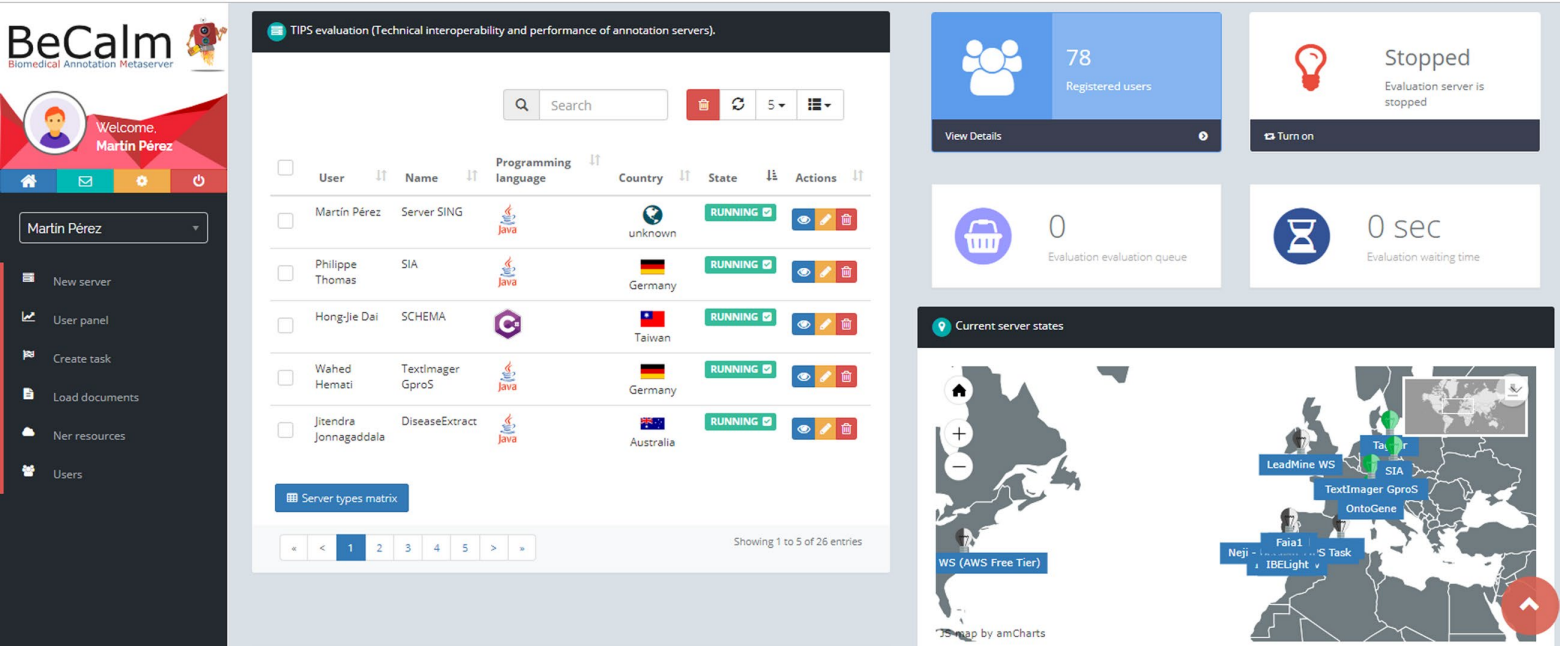
Dashboard of the TIPS organizers in the BeCalm platform. In this dashboard, it is possible to see at any time the status of the different published ASs, the number of registered participants and the status of the metaserver

**Fig. 3:**
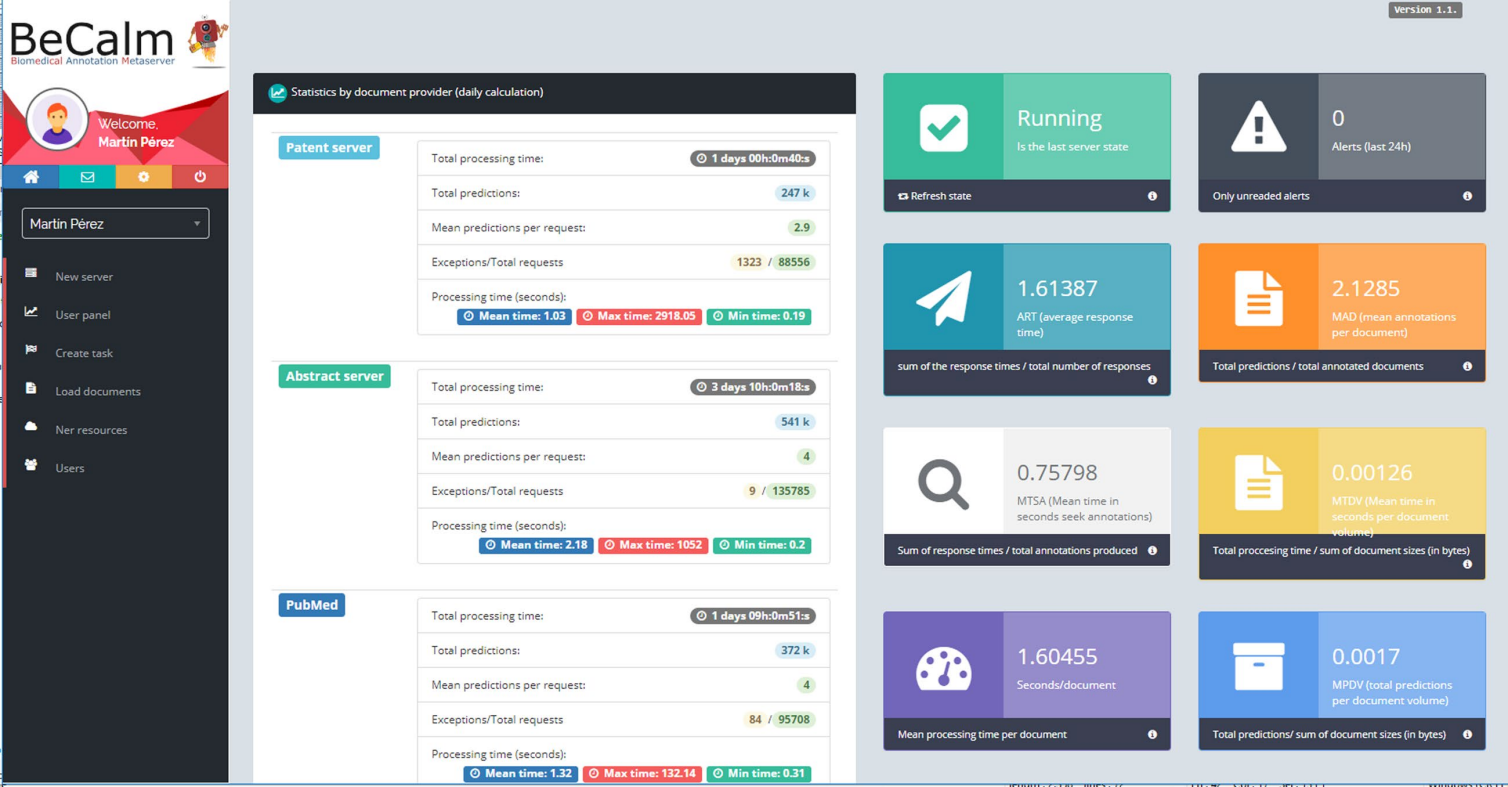
Dashboard of the text mining participant teams in the BeCalm platform for the TIPS track competition. In this dashboard, it is possible to see at any time the state of their ASs along with the number of incidents occurred in communications and an overview of the metrics that the BeCalm metaserver collected to evaluate its performance. In addition, it was possible to observe an AS performance rating for each document server

The BeCalm platform assists the TIPS organizers, namely Martin Krallinger, Anália Lourenço, Martin Pérez-Pérez, Gael Pérez-Rodríguez, Florentino Fdez-Riverola and Alfonso Valencia (Fig. 2), and text mining participant teams (Fig. 3) in doing the registration, testing, debugging and evaluation of the ASs. To do so, BeCalm provided a user-friendly monitoring front-end, that enabled (1) registration of public ASs following a common guideline, (2) the scheduling of annotation/prediction requests to conduct the continuous evaluation, (3) the systematic calculation of server performance metrics, and (4) a detailed log of events about the communication among ASs in order to evaluate the stability.

Due to the nature of the competition, the number of expected responses is the number of requests multiplied by the number of online ASs. Besides, each AS always tries to respond in a short period of time, so a large concurrent number of fast responses is expected. This process of request-response entails that the metaserver must be stable and fully-operative to be able to store and handle the communication in the lowest time possible to guarantee that the AS performance metrics are not affected. To do so, the proposed metaserver structure is a highly efficient solution capable of launching a large number of concurrent requests without interfering with the reception of the responses.

### TIPS first competition and annotation servers

**Fig. 4:**
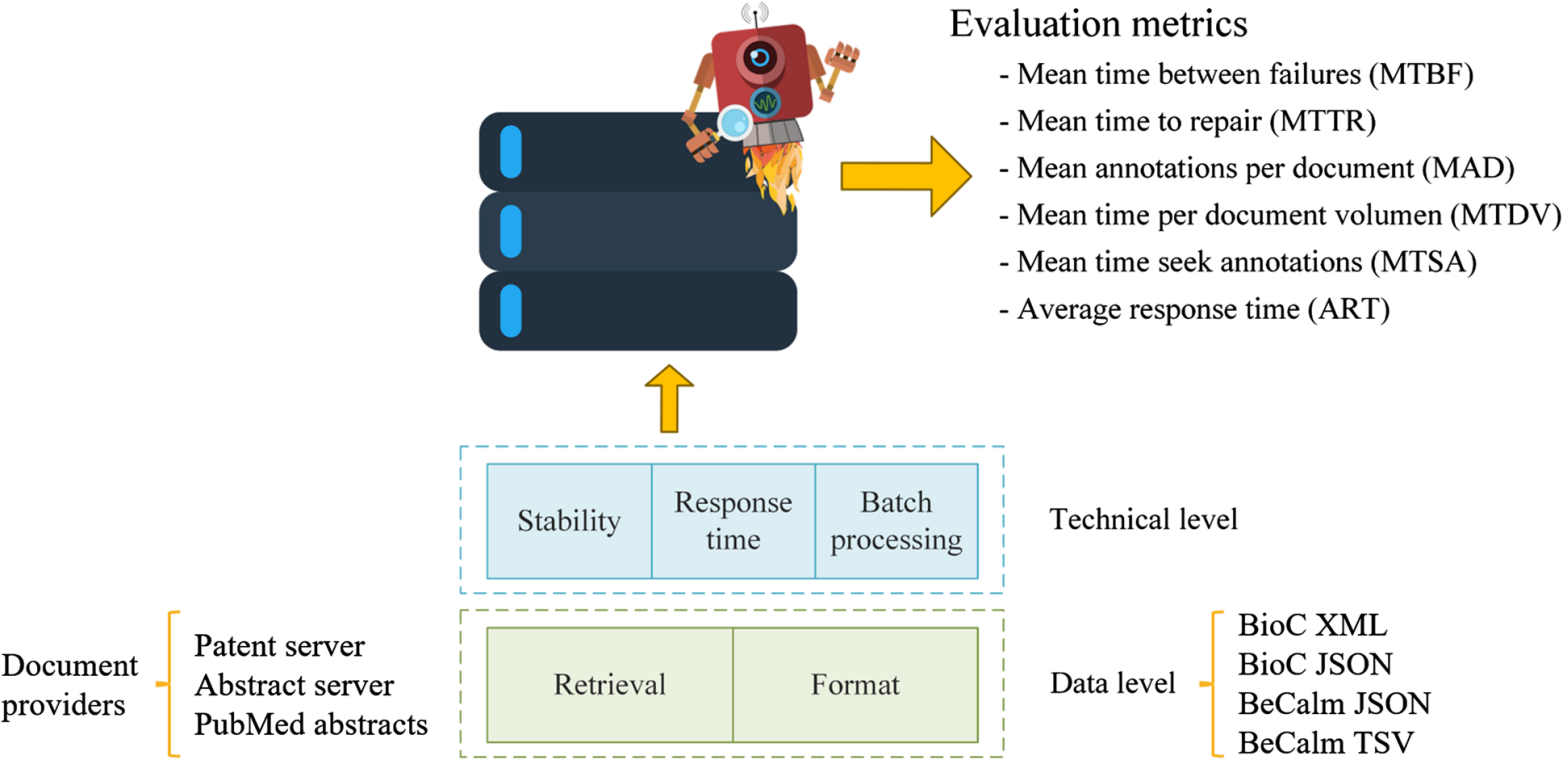
Overview of the general evaluation schema of the TIPS competition

The TIPS evaluation period started on February 5th 2017 and ended March, 30th 2017. This track examined those technical aspects that are critical for making text ASs available in a way that they can be subsequently integrated into more complex text mining workflows by evaluating their performance while serving continuous named entity recognition requests. This more pragmatic and practical view of text ASs was mainly neglected by most other language technology benchmark efforts. The TIPS evaluation setting started by evaluating ASs on the basis of single document requests rather than batch processing of entire multi-document collections. In this line, annotation requests were issued on a regular basis and emulating different daily request loads. The TIPS track was structured into three general levels of evaluation, i.e. data format considerations (interoperability), technical metrics (performance) and functional specifications (Fig. 4).

At the *data level*, evaluation addressed the ability of the ASs to return named entity recognition predictions as structured harmonised data, represented in one or several of the following UTF-8 entity mention character offset specifying formats: XML/BioC, JSON/BioCJSON or TXT/TSV. These supported formats are defined in the API webpage of BeCalm. XML/BioC is a simple format to share text data and annotations and it is widely used in biomedical text mining tasks. All the information related to this format, including the DTD and license, can be checked in its official webpage [32]. The JSON/BioCJSON format is an adaptation of BioC using JSON. Finally, the TXT/TSV is a well-known format previously used in other BioCreative competitions. The structure of this format is tab-based and contains the following columns: document-id, document section, annotation init, annotation end, score, annotation text, entity type, and database id. A complete description of the structure and the restrictions of the supported formats (i.e. DTDs) are accessible at the Additional file 1: Supplementary material 1.

**Fig. 5:**
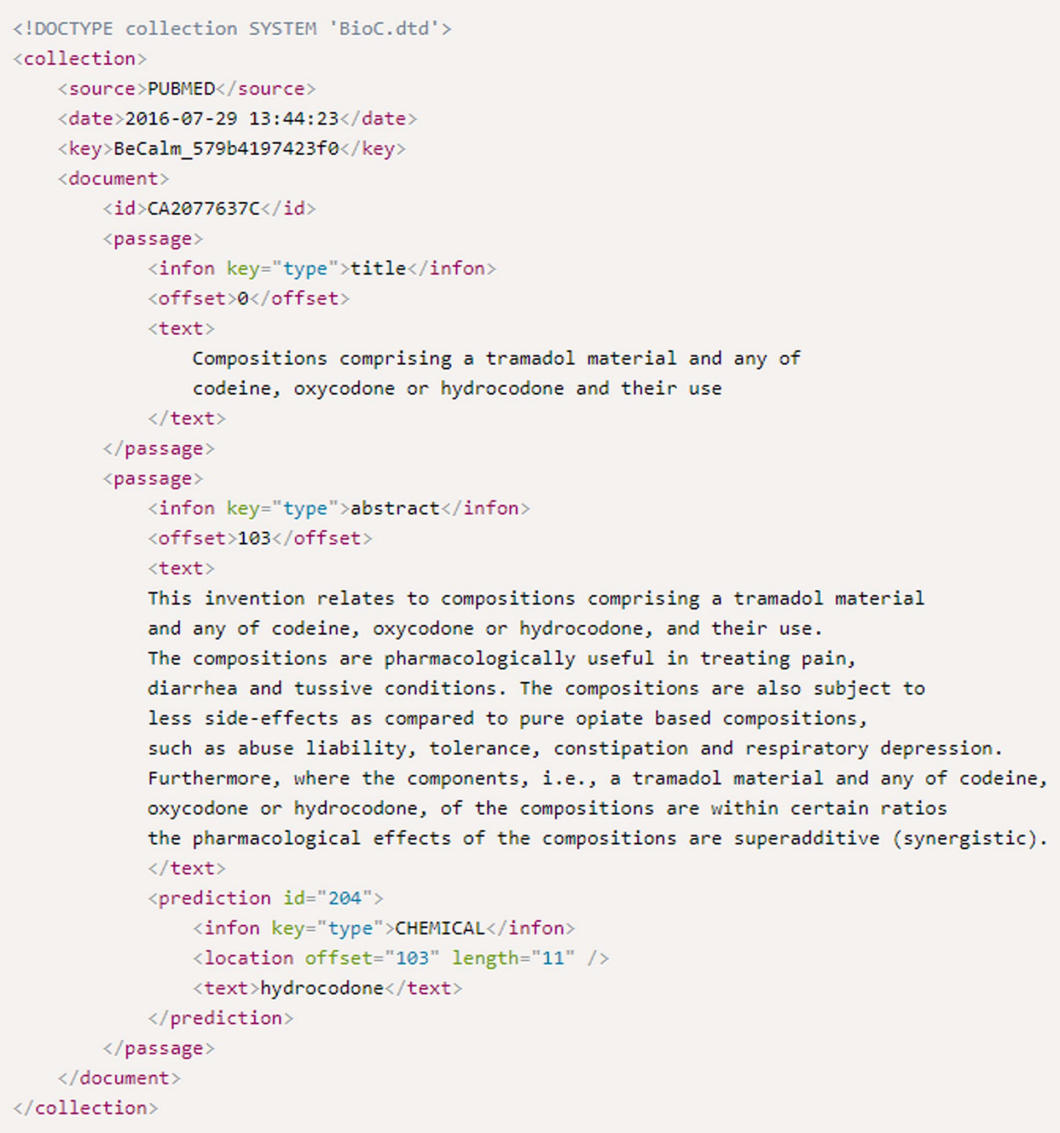
Example of a prediction output in BioC format

Figure 5 shows an example of a prediction output in BioC format. Here, it is possible to observe the document ID (i.e. ID entity), the title of the document (i.e. first passage) and the abstract (i.e. second passage). Inside each passage there are the predicted annotations, in this case, there is only one annotation for the abstract (i.e. prediction entity in the second passage). The entity type, provided in the field “infon”, for the prediction “hydrocodone” represents a chemical (i.e. “hydrocodone” is within the concepts that can be understood as chemical compounds), the initial position of the annotation in the text is “103” characters and the length of the annotation is “13” characters. Using these last values, it is possible to identify the predicted term in the text with independence of text case and format.

In order to examine whether teams were able to cope with heterogeneous types of input documents, TIPS also analysed the ability to retrieve and process documents from different providers, i.e. patents server, abstract server, and PubMed. These document providers, created for the competition, supply the documents in raw text (i.e. without any text style) and in UTF-8 format.

Stability and response time was at the core of technical assessment and constituted the main evaluation metrics used for the TIPS track. Stability metrics were used to characterise the ability of individual servers to respond to continuous requests, to respond within a stipulated time window, and to provide updated server status information. These aspects are key to be able to efficiently exploit and integrate such resources into text mining workflows and to yield a satisfactory user experience. Conversely, response time statistics described the time taken by the ASs to respond to a request, considering the number and the text size of the requested documents as well as the volume of predictions returned. ASs were not allowed to cache the documents, i.e. each document should be downloaded from the specified source upon request. Also, servers should not cache the generated predictions, i.e. each document should be analysed for every request. To test server compliance, some annotation requests included documents (both patents and abstracts) whose contents were randomly modified over time. So, if the set of annotations returned for those documents was identical for all requests that would mean that the server was caching annotations. Finally, the processing of batch requests addressed the ability to respond to requests with a varied number of documents.

The TIPS track guidelines for minimum AS information specification and performance evaluation was aligned with the recommendations of the ELIXIR/EXCELERATE project in benchmarking the ELIXIR catalogue of methods and the OpenMinTeD interoperability specifications [33]. Harmonisation and interoperability were enforced by establishing a minimal set of functional specifications (i.e. mandatory, recommended and optional metadata information). Mandatory metadata included server name, institution/company, server administrator, programming language (main language, if using several), supported biomedical entity annotation semantic types (e.g., chemical entities, genes, proteins, diseases, organisms, cellular lines and types, and mutations), supported annotation formats (e.g., XML/BioC, JSON/BioCJSON or TXT/TSV) and software version. Recommended metadata included software license information, specification of third-party recognition software (if any), dedicated vs. shared server, and relevant references or publications. Optionally, teams could also provide details on the used server operating system, distributed processing, and hardware characteristics (i.e. the number of processors and RAM information).

### TIPS evaluation metrics

Traditional annotation quality evaluation aspects, measured through popular metrics like precision, recall, and balanced F-measure were not examined for the TIPS track evaluation scenario, as those aspects were actually the main focus of other BioCreative tracks, including two sub-tracks (CEMP—chemical entity mention recognition and GPRO—gene and protein related object recognition) also described in this special issue of the Journal of Cheminformatics [34]. The emphasis of the TIPS track assessment was on performance metrics, i.e. reliability indicators and performance indicators. We, therefore, proposed novel evaluation metrics to quantify these aspects when carrying out a comparative analysis of participating web services for biomedical NER. The mean time between failures (MTBF) and the mean time to repair (MTTR) were the key reliability indicators used for TIPS [35, 36]. Conversely, the mean annotations per document (MAD), the mean time per document volume (MTDV), the mean time seek annotations (MTSA), and the average response time (ART) was the key performance indicators examined for this track. Table 1 provides a summary of the used metrics whilst Table 2 provides the equations for the presented metrics. Noteworthy, some of these metrics were inspired by hardware stress testing evaluation scenarios.

**Table 1:** Summary table of the TIPS track evaluation metrics

Name	Description
MTBF	The average elapsed time between AS failures (s)
MTTR	Average time required to repair an AS failure, i.e. the time needed to start the server again after a period of downtime (s)
MAD	The number of annotations per total number of responses (predictions/document)
MTDV	Average time to annotate a document (i.e. answer a request) based on the size of the requested documents (B/s)
MTSA	Average response time considering the number of annotations produced (s)
ART	Average response time (s)

**Table 2:** Equations of the TIPS track evaluation metrics

Name	Equation
MTBF	$\left( {\sum \left( {start\; of\; downtime\left( {failure\; n + 1} \right) - start\; of\; uptime\left( {failure\; n} \right)} \right) } \right)/\left( {number \;of \;failures } \right)$
MTTR	$\left( {\sum \left( {end\; of\; downtime\left( n \right) - start \;of\; downtime\left( n \right)} \right)} \right)/\left( {number \;of \;failures} \right)$
MAD	$\left( {total\; number\; of \;annotations} \right)/\left( {total\; number\; of \;responses} \right)$
MTDV	$\left( {\sum \;response\; time} \right)/\left( {\sum \; document \;size} \right)$
MTSA	$\left( {\sum \;response\; time} \right)/\left( {total \;number \;of\; annotations} \right)$
ART	$\left( {\sum \;response\; time} \right)/\left( {total \;number\; of \;responses} \right)$

## Results

A total of 13 teams participated in TIPS competition and developed 15 different ASs (i.e. teams could present more than one AS). Table 3 shows an overview of the participating teams and their AS (more technical information of the AS are available in Additional file 2: Supplementary Material 2). The participating ASs showed considerable variability in terms of annotation abilities and implementation strategies. Java was clearly the most popular underlying programming language used by participating teams (9 out of 15), nevertheless, some of the servers were implemented in other languages such as C# (2 out of 15), C++, Bash, Python and Crystal (each one was used by 1 participant). Regarding the implementation strategies, most of the participants (9 out of 15) used dictionary-based approaches (exclusively or in combination with other approaches), followed by other strategies like the integration of well-known named entity recognisers (4 out of 15), conditional random fields (3 out of 15) and statistical principle-based (1 out of 15). On the other hand, the used HTTP solution and the type of machine to support the AS during the competition showed less convergence than the previous data. The participants chose technologies like Nginx (2 out of 15), Swagger, Mamba, Jetty, Spring or RabbitMQ (each one was used by 1 participant). Most of the participants mount the ASs in virtual (3 out of 15) and physical (3 out 15) machines. Other alternatives were the usage of Docker containers and cloud infrastructure (each one was used by 1 participant). The ASs that participated in the TIPS track were located worldwide (Europe, Asia, Oceania and America), with major European representation, in particular from Germany and Portugal, as well as teams from Asia (i.e. the Republic of China). The preferred submission format was JSON (11 out of 15), which is becoming more popular lately compared to XML-based annotations. The next most used format was a simple task-specific TSV format specifying the entity offsets (6 out of 15) while, only 3 teams supported BioC submissions, despite the widespread use of this format for BioNLP systems. One of the teams (AS 116) supported all the formats proposed for the TIPS track submissions; while another team (AS 122) offered results in three different output formats (JSON, TSV and BioC). Another team (AS 114) opted for providing server submission in JSON and TSV.

**Table 3:** TIPS teams—annotation server overview

ID	Name	Server contact	Affiliation	Output format	AS location	Programing language	License	Refs
103	SIA*	Philippe Thomas	German Research Center for Artificial Intelligence	JSON	Germany	Java	Apache License 2	[37, 38]
106	LeadMine WS	Daniel Lowe	NextMove Software	JSON	Ireland	Java	–	–
107	SCHEMA	Hong-Jie Dai	National Taitung University	JSON	Republic of China (Taiwan)	C#	–	[39]
108	MRI	Chen-Kai Wang	Taipei Medical University	JSON	Republic of China (Taiwan)	C#	–	[40]
111	DiseaseExtract	Jitendra Jonnagaddala	UNSW Australia	JSON	Australia	Java	Apache License 2	[41]
114	Tagger*	Lars Juhl Jensen	University of Copenhagen	JSON/TSV	Denmark	C++	The BSD 2-Clause ‘Simplified’ or ‘FreeBSD’ License	[42, 43]
116	Neji—BeCalm TIPS Task*	André Santos	IEETA—Institute of Electronics and Informatics Engineering of Aveiro	ALL	Portugal	Java	CC by-nc-sa 3.0	[44, 45]
117	MER*	André Lamúrias	LaSIGE, Faculdade de Ciências, Universidade de Lisboa, Portugal	TSV	Portugal	Bash	MIT	[46, 47]
120	Olelo	Hendrik Folkerts	Hasso Plattner Institute	BioC	Germany	Java	–	[48]
121	LeadMine WS (AWS Free Tier)	Daniel Lowe	NextMove Software	JSON	United States of America	Java	–	–
122	OntoGene*	Lenz Furrer	Institute for Computational Linguistics, University of Zurich	BioC, JSON, TSV	Switzerland	Python	GNU Affero General Public License	[49, 50]
124	TextImager CempS	Wahed Hemati	Text Technology Lab—Goethe-Universität Frankfurt	TSV	Germany	Java	–	[51]
126	TextImager GproM	Wahed Hemati	Text Technology Lab - Goethe-Universität Frankfurt	TSV	Germany	Java	–	[51]
127	READ-Biomed	Read Biomed	University of Melbourne	JSON	Australia	Java/Scala	–	[52]
128	NLProt	Miguel Madrid	Structural Computational Biology Group of the CNIO	JSON	Spain	Crystal	MIT	[53]

The AS location is retrieved from the IP of each AS. Teams that also published a systems description paper in this special issue of the Journal of Cheminformatics is marked by an asterisk

The TIPS track covered a remarkable number of different biomedical entity categories/types, namely the participating ASs enabled the annotation of 12 distinct types. Table 4 provides a summary of the different annotation types returned by each of the participating teams.

**Table 4:** Participating team server NER annotation types

Entity types	Team IDs														
	103	106	107	108	111	114	116	117	120	121	122	124	126	127	128
Chemical (10)		x	x			x	x	x	x	x	x	x	x		
Protein (7)		x					x	x	x	x			x		x
Disease (9)	x	x			x	x	x	x	x	x	x				
Organisms (6)		x				x	x		x		x				x
Anatomical component (4)		x					x		x				x		
Cell line/type (7)		x						x	x	x	x	x	x		
Mutation (4)	x	x					x			x					
Gene (7)		x				x	x		x	x		x	x		
Subcellular structure (7)						x	x	x	x		x	x	x		
Tissue/organ (5)						x	x	x	x						x
miRNA (6)	x			x				x	x			x	x		
GO (1)														x	
Nr. types/team	3	8	1	1	1	6	9	7	10	6	5	5	7	1	3

Chemical compound and Disease entity mention represented the annotation types with greatest server support (i.e. 10 and 9 servers, respectively). Other popular annotation types, covered by 7 servers, were proteins, genes, cell lines/types and subcellular structures. Conversely, GO (i.e. Gene ontology terms) and Mutations, as well as Anatomical structures, were the annotation types with least support (i.e. 1, 4 and 4 servers, respectively). The maximum number of types supported by a single server was 10 (i.e. AS 120), while another server (AS 116) supported also a considerable number of entity types (i.e. 9 types). Besides, 6 out of 15 ASs supported normalization (i.e. link entities to identifiers in biomedical resources). This implies that the TIPS track had enough AS entity types to exploit multiple individual predictions to generate ensemble, consensus or silver standard results for a considerable number of entities. Moreover, when considering the resulting entity co-occurrence relation matrix derived from the various entity types recognised by participating ASs, a total of 66 different bio-entity co-occurrence relation types can theoretically be extracted.

**Fig. 6:**
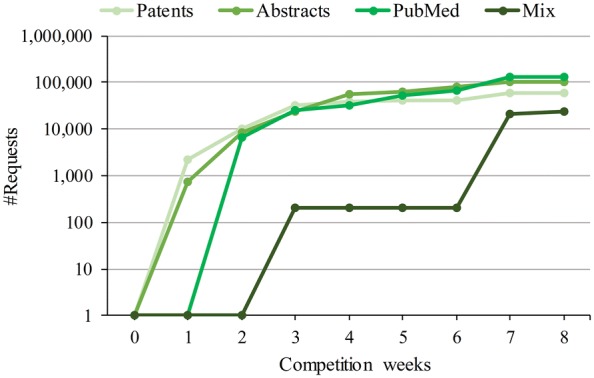
Requests issued per each document provider throughout the evaluation period. Requests are depicted per competition week, from February to March 2017

The core TIPS evaluation period took place during a period of 2 months, from February to March 2017. The aim was to perform a systematic and continuous evaluation of server response under a varied request workload during a certain period of time. Moreover, the schedule comprised requests for three distinct document content providers, i.e. a patent abstract server, a paper abstract server, and PubMed, including a mix of different providers. The average text length of documents from PubMed and Abstract servers were 1326 characters while the average text length of documents from Patents server was 582 characters. Figure 6 shows the time plot covering the competition weeks versus the number of requests launched by each of the content server types. For more information about the processed documents during the TIPS competition see Additional file 3: Supplementary material 3.

Table 5 shows the request workload per month and document provider. Noteworthy, the number of requests sent during the competition comprised regular and irregular time windows and a mixture of document providers. The purpose of this strategy was to emulate periods of low and moderate to high activity with a double objective: (1) it enabled the creation of stress scenarios, which allowed to measure the stability and the behaviour of the ASs under pressure; and (2) it helped the organisers to detect potential caching techniques in the ASs, which were forbidden during the TIPS competition.

**Table 5:** Details on the requests issued during TIPS competition

Doc. provider	Request type	#Requests in February	#Requests in March
Patents	Regular	30,475	1287
Patents	Irregular	9085	20,000
Abstracts	Regular	15,100	30,710
Abstracts	Irregular	8274	45,800
PubMed	Regular	24,710	16,000
PubMed	Irregular	6663	86,325
Mix	Irregular	200	24,000

A significant difference among the response times in high-load request windows compared to homogeneous-load windows may mean that ASs stored the predictions because the communication time between “metaserver-ASs” and “ASs-document provider” was stable.

Table 6 summarises the results of the ASs evaluation. As stated earlier, reliability indicators and performance indicators guided this evaluation. Servers 103, 114, 117, 121 and 127 processed the largest number of requests (i.e. 3.19E+05 requests). Server 120 generated the largest number of predictions (i.e. 2.74E+07 predictions), with an average of 101 predictions per document (i.e. MAD).

**Table 6:** TIPS evaluation data

ID	#Requests	#Predictions	MTSA	MTDV	MAD	ART	MTBF	MTTR
103	***3.19E+05***	6.70E+05	7.58E−01	1.32E−03	2.13E+00	1.61E+00	***4.58E+06***	***0.00E+00***
106	3.12E+05	4.07E+06	8.59E−02	9.42E−04	1.34E+01	1.15E+00	***4.58E+06***	***0.00E+00***
107	2.95E+05	1.14E+06	2.85E+02	1.00E+00	4.27E+00	1.22E+03	4.62E+05	2.23E+05
108	1.23E+05	0.00E+00	–^a^	3.03E−02	0.00E+00	3.63E+01	***4.58E+06***	***0.00E+00***
111	3.11E+05	5.59E+05	3.55E+02	6.48E−01	2.27E+00	8.06E+02	5.19E+05	2.12E+04
114	***3.19E+05***	4.78E+06	1.21E−01	1.48E−03	1.51E+01	1.82E+00	***4.58E+06***	***0.00E+00***
116	2.29E+05	2.31E+06	3.83E+02	7.55E+00	2.35E+01	9.01E+03	8.11E+04	4.65E+05
117	***3.19E+05***	7.13E+06	1.29E−01	2.38E−03	2.25E+01	2.90E+00	***4.58E+06***	***0.00E+00***
120	2.91E+05	***2.74E+07***	***1.37E−02***	1.15E−03	***1.01E+02***	1.39E+00	***4.58E+06***	***0.00E+00***
121	***3.19E+05***	3.30E+06	1.18E−01	9.96E−04	1.04E+01	1.22E+00	***4.58E+06***	***0.00E+00***
122	3.16E+05	4.42E+06	7.23E−02	***8.58E−04***	1.48E+01	***1.07E+00***	***4.58E+06***	***0.00E+00***
124	4.98E+04	2.98E+04	1.55E+01	4.49E−02	3.29E+00	5.14E+01	1.17E+06	6.09E+04
126	4.98E+04	3.22E+04	1.50E+01	5.00E−02	3.69E+00	5.58E+01	5.86E+05	8.98E+04
127	***3.19E+05***	2.79E+06	4.20E−01	3.07E−03	8.90E+00	3.74E+00	***4.58E+06***	***0.00E+00***
128	1.87E+05	8.57E+05	5.44E+02	6.35E+00	1.38E+01	7.52E+03	1.73E+05	1.47E+05

Bolditalic data represents the top values for each metric

^a^This server provided empty prediction files for all requests

Server 120 took an average time of 0.013 s to produce a prediction (i.e. MTSA). The minimum processing time value (i.e. ART) was 1.07 s, and the minimum processing time per document volume (i.e. MTDV) was 8.58E−04 bytes/s (i.e. server 122). During the TIPS competition, 9 servers operated uninterrupted. Among the rest, the server 111 had the smallest recovering score (i.e. MTTR) restarting after 5.8 h.

## Discussion

It is remarkable that most of the participating servers showed great reliability and stability through the TIPS evaluation phase. For example, for a total of 4,092,502 requests, the median response time for most servers was below 3.74 s, with a median of 10 annotations per document. In terms of document providers, the median response time was 2.85 s for the patent server and 3.01 s for the abstract server. The PubMed content server case showed slightly higher response times (3.48 s per request), which can be explained by the need of retrieving these abstracts upon request, i.e. strictly depending on PubMed service and without any local caching. We have explored with the responsible of Europe PMC whether a specific server devoted to such community challenges would be necessary for future challenges, in order to not interfere with the regular content providing servers used for bibliographic searches. In fact, Europe PMC expressed interest in the potential integration of participating ASs into text mining workflows. Moreover, we foresee that future shared tasks building on TIPS should directly involve content providers, publishers or aggregators to distribute content in the form of especially devoted document servers, while a metaserver like BeCalm would serve as a sort of broker and registry communicating between the content servers and participating ASs.

Most servers were able to process 100,000 requests, for different providers, in 5 days. Considering that many participants stated that their servers could perform batch processing, the obtained results are very promising, as through batch processing the volume of processed documents could easily grow to one million records.

While the quality of the annotations was not part of the evaluation, it was interesting to inspect the methodology and implementation strategy proposed by the different servers. Most of the times, the ASs used dictionary look-up and/or machine learning methods (e.g. conditional random fields) to perform named entity recognition. In particular, the Gene Ontology [54], Cellosaurus [55], miRBase [56], UMLS [57], ChEBI [58] and ChEMBL [59] were some of the most used database sources. On the contrary, other participants (e.g. team 128 using the NLProt tagger) had to refactor the original pipeline of particular well-known NER systems.

Currently, 6 out of 15 ASs provide normalized or grounded entity mentions, returning not only mention offsets but also their corresponding concept or database identifiers. In the future, it would be interesting to allow settings where the mention recognition modules and the normalization of these mentions to concept identifiers are de-coupled, in order to promote systems that are specialized in either of these two tasks. Other aspects that should be explored in more detail for future efforts following the TIPS track include the systematic generation of lexical resources and name gazetteers through the results obtained by the ASs. Manual validation or curation of lexical resources generated by ASs can, in turn, be used to improve the original look-up approaches.

Consensus mentions based on multiple predictions generated by different ASs were examined by the original BioCreative Metaserver (BCMS) but was not examined in detail for TIPS. The creation of optimal consensus predictions that combine aspects related to both quality and technical performance would definitively be worthwhile to be explored by future community evaluation efforts. Moreover, this also implies the exploration of the current need to visualize the results into a single interface or to empower user interaction to select certain outputs, ASs or combinations thereof.

Noteworthy, the number of supported annotation types was relevant for TIPS evaluation, because the MTSA value (i.e. the average response time based on the number of annotations produced) was lower for servers supporting multiple types whereas the MAD value (i.e. the number of annotations per total number of documents) was higher. Typically, the number of predictions grew in proportion with the number of supported types, i.e., the greater the number of supported annotation types, the greater the number of predictions returned per request. So, the metrics proposed for this first experimental task should be viewed only as illustrative of the performance of the ASs.

Modularise severs for each annotation type, that is, the purpose was not to deem an AS as being superior because it showed better results in one specific metric. In fact, these metrics should be considered as a whole and their practical utility lays on providing knowledge to enhance or fine-tune annotation services according to different usage requirements.

There have been concerns related to some limitations associated with the use of web services in terms of (1) reproducibility, as services might change over time or even become unavailable, (2) end users can not directly inspect the underlying code which makes debugging difficult and (3) they cannot be directly exploited with the data to be processed is sensitive or has copyright issues. There are also mitigations that can be adopted to mitigate these potential downsides of web-services, through the use of components with a service API (microservices), portable packaging and dockerization. Efforts like the OpenMinTeD platform has shown that dockerized web-services can be smoothly integrated into more complex text processing workflows.

## Conclusions

The BeCalm TIPS task was a novel experimental task that systematically evaluated the technical performance aspects of online entity recognition systems. It raised the interest of a significant number of participants. Also noteworthy, many of the ASs were built on the shoulders of systems that participated in prior BioCreative competitions that focussed on quality aspects.

Future editions of the TIPS competition will address the ability to process documents in bulk as well as to annotate full-text documents. In addition, feedback obtained from the participants is being considered, e.g. using the median or modal time values instead of the average time to avoid sporadic high response times. Hopefully, the evaluated tools may constitute valuable public building blocks for biomedical applications. In particular, such building blocks could be of help in the extraction of relevant associations of biomedical concepts (e.g. chemical-gene interactions or disease mutation interactions). Indeed, the TIPS task aims to promote the development and research of new online text mining tools of practical use. Future efforts, following the settings already explored by TIPS, should also go beyond the processing of textual data in English and include additional document types as well as data in other languages. Efforts like the Spanish Plan for the Advancement of Language Technology is particularly interested in promoting competitive evaluation tasks that do examine also technical and performance aspects of components, to shorten the path between academic language technology developments and their exploitation by commercial initiatives.

## Additional files

**Additional file 1.** Description of the structure and the restrictions of the supported formats.

**Additional file 2.** Technical information of the Annotation Servers.

**Additional file 3.** Processed document IDs during the TIPS competition.

## Abbreviations

AS: annotation server
ASs: annotation servers
TIPS: technical interoperability and performance of annotation servers
REST: representational state transfer
API: application programming interface
MTBF: mean time between failures
MTTR: mean time to repair
MAD: mean annotations per document
MTDV: mean time per document volume
MTSA: mean time seek annotations
ART: average response time

## Availability of data and materials

The DTDs of the supported formats for exchange predictions, a table with the technical information about the participants ASs (including a reference to the publications) and the IDs used during TIPS competition are presented as supplementary material. Plus, in the following URL, it is presented an example to construct an AS: https://github.com/abmiguez/dummyServer.
