# Supplementary material for: Next generation community assessment of biomedical entity recognition web servers: metrics, performance, interoperability aspects of BeCalm
Source: J Cheminform. 2019 Jun 24;11:42. doi: 10.1186/s13321-019-0363-6 (PMC6591930; doi:10.1186/s13321-019-0363-6)
Supplement: Supplementary file 2 — Additional file 2. Technical information of the Annotation Servers. [file 13321_2019_363_MOESM2_ESM.docx]

# Supplementary material 2 – Technical information of the Annotation Servers

| **ID** | **Name** | **Output format** | **HTTP solution** | **System design** | **AS machine** | **Programing language** | **Support normalization** | **License** | **Repository** | **Refs** |
| --- | --- | --- | --- | --- | --- | --- | --- | --- | --- | --- |
| 103 | SIA | JSON | RabbitMQ | Dictionary-based, Implementing NERs | - | Java | No | Apache License 2 | [1] | [2, 3] |
| 106 | LeadMine WS | JSON | - | Dictionary-based | - | Java | Yes | - | - | - |
| 107 | SCHEMA | JSON | Apache Spark | Conditional random fields | Virtual | C# | No | - | - | [4] |
| 108 | MRI | JSON | - | Statistical principle-based approach | - | C# | No | - | - | [5] |
| 111 | DiseaseExtract | JSON | Swagger | Conditional random fields | Physical | Java | Yes | Apache License 2 | [6] | [7] |
| 114 | Tagger* | JSON /TSV | Mamba | Dictionary-based | Shared-physical | C++ | Yes | The BSD 2-Clause 'Simplified' or 'FreeBSD' License | [8] | [9, 10] |
| 116 | Neji - BeCalm TIPS Task* | ALL | Jetty | Dictionary-based, Conditional random fields | Docker | Java | No | CC by-nc-sa 3.0 | [11] | [12, 13] |
| 117 | MER* | TSV | Nginx | Dictionary-based | Cloud infrastructures | Bash | No | MIT | [14] | [15, 16] |
| 120 | Olelo | BioC | Spring | Dictionary-based | Physical | Java | No | - | - | [17] |
| 121 | LeadMine WS (AWS Free Tier) | JSON | - | Dictionary-based | - | Java | Yes | - | - | - |
| 122 | OntoGene* | BioC, JSON, TSV | Nginx | Dictionary-based | Virtual | Python | Yes | GNU Affero General Public License | [18] | [19, 20] |
| 124 | TextImager CempS | TSV | TextImager Orchestator | Implementing NERs | - | Java | No | - | - | [21] |
| 126 | TextImager GproM | TSV | TextImager Orchestator | Implementing NERs | - | Java | No | - | - | [21] |
| 127 | READ-Biomed | JSON | Node.js HTTP server | Dictionary-based | - | Java/Scala | No | - | - | [22] |
| 128 | NLProt | JSON | Kemal | Implementing NERs | Virtual | Crystal | Yes | MIT | - | [23] |

# References

1. Kirschnick J (2019) Scalable Interoperable Annotation Server (SIA). https://github.com/Erechtheus/sia. Accessed 23 Apr 2019

2. Wynn R, Oyeyemi SO, Johnsen J-AK, Gabarron E (2017) Tweets are not always supportive of patients with mental disorders. Int J Integr Care 17:149. https://doi.org/10.5334/ijic.3261

3. Kirschnick J, Thomas P, Roller R, Hennig L (2018) SIA: a scalable interoperable annotation server for biomedical named entities. J Cheminform 10:63. https://doi.org/10.1186/s13321-018-0319-2

4. Dai H-J, Rosa MAC dela, Zhang D, et al (2017) NTTMU-SCHEMA BeCalm API in BioCreative V . 5. In: Proceedings of the BioCreative V.5 Challenge Evaluation Workshop. Barcelona, pp 196–204

5. Wang C-K, Dai H-J, Chang N-W (2017) Micro-RNA Recognition in Patents in BioCreative V . 5. In: Proceedings of the BioCreative V.5 Challenge Evaluation Workshop. Barcelona, pp 205–210

6. (2019) Disease Extract. https://github.com/TCRNBioinformatics/DiseaseExtract. Accessed 23 Apr 2019

7. Jonnagaddala J, Dai H-J, Wang C-K, Lai P-T (2017) Performance and interoperability assessment of Disease Extract Annotation Server ( DEAS ). In: Proceedings of the BioCreative V.5 Challenge Evaluation Workshop. Barcelona, pp 156–162

8. Juhl-jensen L (2019) Tagger. https://bitbucket.org/larsjuhljensen/tagger/. Accessed 23 Apr 2019

9. Jensen LJ (2017) Tagger: BeCalm API for rapid named entity recognition. In: Proceedings of the BioCreative V.5 Challenge Evaluation Workshop. Barcelona, pp 122–129

10. Pletscher-Frankild S, Jensen LJ (2019) Design, implementation, and operation of a rapid, robust named entity recognition web service. J Cheminform 11:19. https://doi.org/10.1186/s13321-019-0344-9

11. Campos D (2017) Neji. https://github.com/BMDSoftware/neji. Accessed 23 Apr 2019

12. Santos A, Matos S (2017) Neji : DIY web services for biomedical concept recognition. In: Proceedings of the BioCreative V.5 Challenge Evaluation Workshop. Barcelona, pp 54–60

13. Matos S (2018) Configurable web-services for biomedical document annotation. J Cheminform 10:68. https://doi.org/10.1186/s13321-018-0317-4

14. Lamurias A (2019) MER (Minimal Named-Entity Recognizer). https://github.com/lasigeBioTM/MER. Accessed 23 Apr 2019

15. Couto FM, Campos L, Lamurias A (2017) MER: a Minimal Named-Entity Recognition Tagger and Annotation Server. In: Proceedings of the BioCreative V.5 Challenge Evaluation Workshop. Barcelona, pp 130–137

16. Couto FM, Lamurias A (2018) MER: a shell script and annotation server for minimal named entity recognition and linking. J Cheminform 10:58. https://doi.org/10.1186/s13321-018-0312-9

17. Folkerts H, Neves M (2017) Olelo’s named-entity recognition web servicein the BeCalm TIPS task. In: Proceedings of the BioCreative V.5 Challenge Evaluation Workshop. Barcelona, pp 167–174

18. Furrer L (2019) OGER: OntoGene’s Biomedical Entity Recogniser. https://github.com/OntoGene/OGER. Accessed 23 Apr 2019

19. Furrer L, Rinaldi F (2017) OGER: OntoGene’s Entity Recogniser in the BeCalm TIPS Task. In: Proceedings of the BioCreative V.5 Challenge Evaluation Workshop. Barcelona, pp 175–182

20. Furrer L, Jancso A, Colic N, Rinaldi F (2019) OGER++: hybrid multi-type entity recognition. J Cheminform 11:7. https://doi.org/10.1186/s13321-018-0326-3

21. Hemati W, Uslu T, Mehler A (2017) TextImager as an interface to BeCalm. In: Proceedings of the BioCreative V.5 Challenge Evaluation Workshop. Barcelona, pp 163–166

22. Teng R, Verspoor K (2017) READ-Biomed-Server : A Scalable Annotation Server Using the UIMA Concept Mapper. In: Proceedings of the BioCreative V.5 Challenge Evaluation Workshop. Barcelona, pp 183–190

23. Madrid MA, Valencia A (2017) High-throughput , interoperability and benchmarking of text-mining with BeCalm biomedical metaserver. In: Proceedings of the BioCreative V.5 Challenge Evaluation Workshop. Barcelona, pp 146–155
